# ^1^H-MRS brain metabolites as biomarkers of high-altitude hypobaric hypoxia following mild traumatic brain injury in mice

**DOI:** 10.3389/fnins.2026.1808567

**Published:** 2026-05-14

**Authors:** Young Woo Park, Alexandru Korotcov, Asamoah Bosomtwi, Nathan P. Cramer, Xiufen Xu, Dinesh K. Deelchand, Małgorzata Marjańska, Zygmunt Galdzicki

**Affiliations:** 1 The Henry M. Jackson Foundation for the Advancement of Military Medicine, Inc. (HJF), Bethesda, MD, United States; 2 Department of Radiology and Bioengineering, School of Medicine, Uniformed Services University of the Health Sciences, Bethesda, MD, United States; 3 Georgia Cancer Center, Augusta University, Augusta, GA, United States; 4 Department of Anatomy, Physiology and Genetics, School of Medicine, Uniformed Services University of the Health Sciences, Bethesda, MD, United States; 5 Department of Anatomy and Neurobiology, University of Maryland School of Medicine, Baltimore, MD, United States; 6 Department of Radiology, Center for Magnetic Resonance Research, University of Minnesota, Minneapolis, MN, United States; 7 Neuroscience Graduate Program, School of Medicine, Uniformed Services University of the Health Sciences, Bethesda, MD, United States

**Keywords:** ^1^H-MRS (proton magnetic resonance spectroscopy), closed-head injury, high altitude metabolism, concussion, high altitude adaptation, high altitude hypoxia, mTBI (mild traumatic brain injury), preclinical MRS

## Abstract

**Introduction and objective:**

Populations at high altitude (HA) face a higher incidence and severity of traumatic brain injury (TBI). This pilot study utilized longitudinal ^1^H-MRS to identify neurochemical biomarkers of HA adaptation and the subsequent metabolic response to mild TBI (mTBI).

**Methods:**

Male C57BL/6J mice were exposed to simulated HA (5,000 m) or sea level (SL) for 12 weeks. Following adaptation, a unilateral mTBI was induced via closed head injury (CHI). Mice were then monitored for an additional 2 weeks at HA (total duration of 14 weeks). *In vivo* ^1^H-MRS spectra (7 T) were collected from the frontal cortex, hippocampi, and cerebellum at weeks 0, 4, 12 to assess HA adaptation. Following the CHI, subsequent measurements were collected at week 12 (post-injury) and week 14 to monitor longitudinal neurochemical responses to the mTBI.

**Results:**

Chronic HA exposure induced significant reductions in myo-inositol (Ins) and total choline (tCho) in the hippocampus, establishing a baseline of metabolic fragility that sensitized the brain to subsequent traumatic insult. Post-mTBI, the HA group exhibited a profound “metabolic crisis,” characterized by significantly lower tCho and failed recovery of total N-acetylaspartate (tNAA) compared to SL controls. Total creatine (tCr) was the most acutely affected metabolite, underscoring a depletion of the bioenergetic reserve.

**Conclusion:**

Chronic hypobaric hypoxia fundamentally alters baseline brain metabolism and impairs the neurochemical recovery from mTBI. These findings suggest that standard recovery protocols may be insufficient for HA-adapted populations and highlight ^1^H-MRS as a critical tool for detecting “invisible” metabolic vulnerability in extreme environments.

## Introduction

Mild traumatic brain injury (mTBI), commonly referred to as concussion, represents the most prevalent form of traumatic brain injury (TBI) (Shaw, 2002). It is characterized by impaired cognitive function and a constellation of acute symptoms, including headache, nausea, mental fog, and irritability (Silverberg et al., 2023). Because mTBI can occur in diverse settings, understanding how environmental stressors influence its effects is critical. One such stressor is exposure to high altitude (HA), where hypobaric and hypoxic conditions can disrupt physiological mechanisms in the brain (Cramer et al., 2019) and may exacerbate neurological vulnerability as evidenced by a recent epidemiological study (Rauch et al., 2021) indicating a higher incidence of TBI at HA, as well as more severe neurological consequences compared to similar injuries sustained at sea level (SL) (Li A. et al., 2022; Yang et al., 2022). Populations living or working at HA, including special athletes, military personnel, and astronauts, are therefore at heightened risk, yet the mechanisms underlying this interaction remain poorly understood.

Experimental models provide further evidence of altitude-related vulnerability. In mice, our group has demonstrated that subacute (≤ 3 weeks) and chronic (≥ 8 weeks) exposures impair contextual fear memory recall and novel object recognition (12 weeks) (Cramer et al., 2019; Hatch et al., 2024; Sharma et al., 2019). Prolonged HA exposure (1–6 months) resulted in abnormal magnetic resonance image (MRI) measurements, including cerebral blood flow (CBF), T_2_ relaxation time in both gray and white matter, and diffusion tensor imaging (DTI) metrics across the time period. Furthermore, we have demonstrated that HA exposure significantly elevates inflammatory biomarkers and promotes microglial activation, while concurrently impairing cerebral glucose metabolism and hippocampal synaptic plasticity (Cramer et al., 2019; Hatch et al., 2024). These results indicate potential neurovascular and white matter remodeling (Cramer et al., 2019; Hatch et al., 2024), and align with the prior reports of neurobehavioral deficits and neurovascular/neuroinflammatory pathology of chronic hypobaric hypoxia in rodent (Alam et al., 2019; Dheer et al., 2018; Skovira et al., 2016) and human models (Chen et al., 2016a; Chen et al., 2016b; Hackett et al., 2019).

Our prior work on mTBI using a repetitive closed head injury model indicates that such injury induces persistent neurophysiological changes, disrupting excitation/inhibition balance and leading to functional deficits in synaptic plasticity (Logue et al., 2016; Marion et al., 2018). Regions such as the hippocampus appear to exhibit hyperexcitability that resulted in deficits of contextual fear memory recall relative to sham controls. In corpus callosum, temporal changes in axonal myelination and function were shown with slowing of action potential velocity 3 days after injury. Upon further observation, mice seem to recover from this phenomenon after 2 weeks (Logue et al., 2016), but significant apoptosis of oligodendrocytes and atrophy of the corpus callosum was evident after 6–8 weeks (Marion et al., 2018; Marion et al., 2019). These findings underscore region-specific and time-dependent patterns of injury progression.

In our recent longitudinal neuroimaging study we have shown specific changes related to mTBI in the context of HA in CBF, T_2_ values, DTI, positron emission standardized uptake values and global brain volume (Browne et al., 2026). Histopathological evaluation and protein expression assessments suggest an interaction of HA and mTBI on microglial activation. This study suggests that mTBI at altitude exacerbates disruption of white matter integrity and glial function, indicating a synergistic impact of HA and mTBI on neurovascular integrity and immune function in a non-linear manner within specific brain regions.

Chronic HA exposure induces complex neurovascular adaptations and the structural and behavioral phenotypes suggest that despite vascular remodeling, the brain remains in a state of metabolic fragility. This concept aligns with the “window of metabolic brain vulnerability” proposed in concussive injury, where perturbations in oxidative metabolism and ionic homeostasis render neural tissue hypersensitive to secondary insults (Vagnozzi et al., 2007). Specifically, traumatic injury triggers a cascade involving ionic shifts, indiscriminate glutamate release, and a hyperglycolytic phase followed by prolonged metabolic depression (Lyons et al., 2018; Vagnozzi et al., 2013). In the context of HA, where baseline hypoxia already compromises mitochondrial function and upregulates hypoxia-inducible factors (Li Y. et al., 2022), the metabolic reserve and microglia neuroprotective/repair abilities required to recover from mTBI may be critically depleted. While structural and functional effects of HA and mTBI have been reported, the neurochemical metabolic interactions that occur at the molecular level remain poorly understood.

Proton magnetic resonance spectroscopy (^1^H-MRS) is a powerful tool to detect neurochemical changes and alterations in brain metabolism in response to external stressors. Therefore, characterizing the neurochemical profile in HA and HA-TBI via ^1^H-MRS is essential to bridge the gap between the gross morphological changes observed in MRI/DTI and the functional deficits observed in behavior. In this pilot study, we investigated neurophysiological adaptations to HA by monitoring regional brain metabolites longitudinally with ^1^H-MRS in mice following chronic exposure to a simulated HA environment of 5,000 m elevation (equivalent to inspired PO_2_ of 78 mmHg, 10–11% O_2_, ∼ 60% SpO_2_) for 12 weeks. We then examined how these HA-impacted metabolites responded to unilateral mTBI, induced using our established preclinical mouse mTBI model (Logue et al., 2016), over a 14-day period.

## Methods

### Study design

**FIGURE 1:**
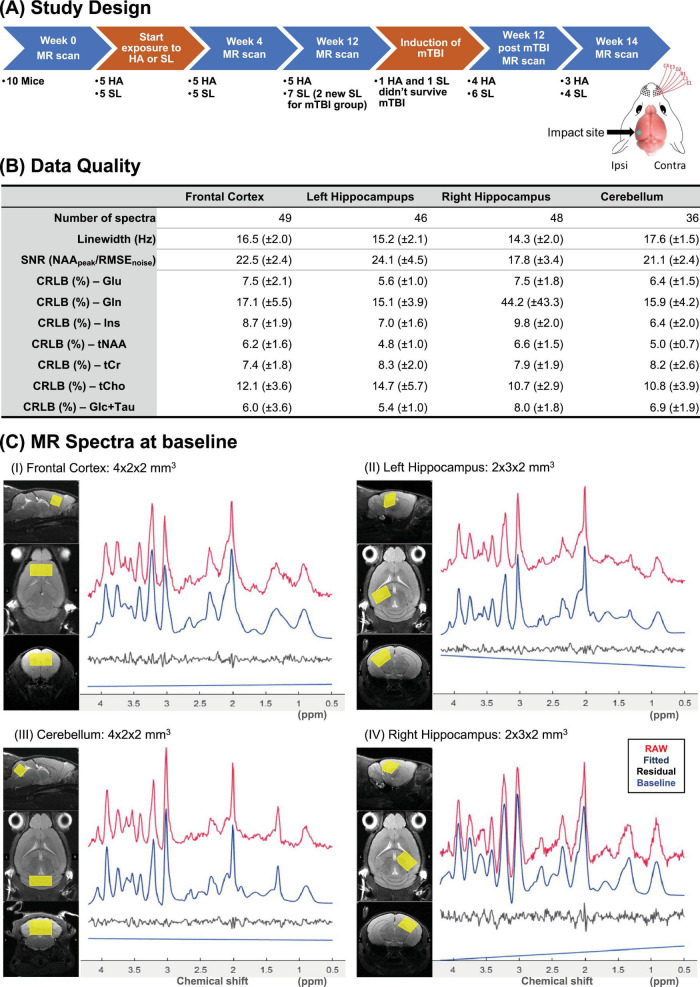
**(A)** Experimental timeline illustrating key interventions (orange) and measurement times (blue). The number of animals assessed at each time point and the equipment used for interventions are also shown. **(B)** Overview of MRS quality metrics shown as mean (± standard deviation). **(C)** Sample MR spectra at baseline and LCModel outputs from (I) frontal cortex, (II) left hippocampus, (III) cerebellum, and (IV) right hippocampus, shown alongside corresponding volume-of-interest placements on T_2_-weighted images. SNR, signal-to-noise ratio; Linewidth, full-width-half-maximum of water; CRLB, Cramér–Rao lower bounds; Glu, glutamate; Gln, glutamine; Ins, myo-Inositol; tNAA, total NAA (sum of NAA and NAAG); tCr, total creatine (sum of Cr and PCr); tCho, total choline (sum of PCho and GPC); Glc + Tau, sum of glucose and taurine.

The study protocol was reviewed and approved by the institutional animal care and use committee (IACUC) at the Uniformed Services University of the Health Sciences prior to the commencement. Figure 1A illustrates the experimental timeline. Ten eight-week-old male C57BL/6J mice (Jackson laboratories) initially housed at SL underwent baseline MR scans. Male mice were used in this study to limit variables impacting outcomes to just that of HA and mTBI and sex as a biological variable was not evaluated in this study.

Mice assigned to the HA group (randomly split into SL and HA group) were then placed in a hypobaric chamber (Reimers System, Lorton, United States) simulating 5,000 m elevation as described in Cramer et al. (2019) and Hatch et al. (2024). Exposure lasted 12 weeks, with weekly re-pressurization to SL for cage maintenance. Ascent and descent were controlled at 200 m/min to ensure adequate mice acclimatization. Given the exploratory nature of this preclinical 7 T ^1^H-MRS study and prior rodent mTBI MRS investigations demonstrating detectable neurometabolic alterations with group sizes of approximately 4–6 animals (Vagnozzi et al., 2007), a sample size of 5 animals per group was used; however, this modest cohort represents a limitation that may reduce statistical power and sensitivity to subtle effects, and limit generalizability of the findings.

Following the exposure period, mice were anesthetized with isoflurane and subjected to unilateral mTBI via three episodes of closed head injury (CHI) (Logue et al., 2016) that were spaced 24 hours apart. CHI was induced using an electromagnetically controlled cortical impact device (Leica Impact One™ Stereotaxic Impactor, Buffalo Grove, United States), targeting a site near Bregma (X = 3.0 mm lateral, *Y* = −2.0 mm posterior, *Z* = 1.5 mm depth, left hemisphere). Impact parameters included a 5 mm tip, 4 m/s velocity, 15°-angle, and 100 ms dwell time. After each CHI, mice were returned to their assigned chambers.

### MRS data acquisition

MR imaging was performed with a 7T Bruker Biospec 70/20 system (Billerica, United States) running ParaVision 6.0.1 software. A radiofrequency coil with birdcage transmit and a 4-channel receive array elements was used. All animals were anesthetized with a mixture of isoflurane/medical air during the scan with their respiration rate and rectal temperature continuously monitored (SA-instruments, Stony Brook, NY) to ensure their wellbeing. MR scans were performed at the baseline time point (Week 0), weeks 4 and 12 of HA exposure, following the completion of 3 episodes of CHI at week 12 and again at week 14 of HA exposure for total of 5 time points for each animal. The first 3 time points used to assess the effect of HA and the latter 3 used to evaluate the changes following mTBI. The timeline was specifically designed to ensure the mTBI was superimposed on a brain already fully adapted to chronic hypobaric hypoxia, allowing us to assess injury response in a state of established “metabolic fragility”.

Spectra were acquired using a vendor-supplied PRESS (Bottomley, 1987) sequence (TR/TE = 2,500/16.5 ms, 256 transients, receiver bandwidth = 3,301 Hz) from four volumes-of-interest (VOIs): frontal cortex (4 × 2 × 2 mm^3^), left hippocampus (2 × 3 × 2 mm^3^), right hippocampus (2 × 3 × 2 mm^3^), and cerebellum (4 × 2 × 2 mm^3^). VOI placement was guided by multi-echo T_2_-weighted RARE images (TR/TE = 4,000/10–110 ms; RARE factor = 2; 4 averages; scan time = 15 min). Vendor-supplied MAPSHIM shimming was used to correct for B_0_ inhomogeneity within the VOIs. Full width at half maximum (FWHM) of the water reference spectrum, obtained along with the metabolite spectrum, was used as a quality control measure for B_0_ inhomogeneity correction with 20 Hz set as a cutoff threshold.

### Data analysis

Each spectrum was processed using MATLAB (Natick, United States) and MRspa toolbox (Deelchand, 2020) for frequency, phase, and eddy-current corrections. Metabolite quantification was performed with LCModel (Provencher, 2001) (v6.3.1N) using a simulated basis set, which was simulated via density-matrix formalism using known chemical shifts and J-coupling constants (Govindaraju et al., 2000; Kaiser et al., 2010) and the same RF pulse shapes, duration and inter-pulse delays as used in the PRESS sequence for measurement. The simulated basis set comprised alanine (Ala), ascorbate (Asc), aspartate (Asp), creatine (Cr), γ-aminobutyric acid (GABA), glucose (Glc), glutamine (Gln), glutamate (Glu), glycerylphosphorylcholine (GPC), glutathione (GSH), *myo*-inositol (Ins), *scyllo*-inositol (sIns), lactate (Lac), phosphoethanolamine (PE), phosphocholine (PCho), phosphocreatine (PCr), *N*-acetylaspartate (NAA), *N*-acetylaspartylglutamate (NAAG), and taurine (Tau). We used LCModel’s built-in simulated peaks setting for fitting macromolecules (Mac).

We report metabolites levels for those with high fitting confidence (mean Cramér–Rao lower bounds, CRLB, of 20% or less, computed by LCModel). Values of two metabolites were summed if they showed negative correlations (*r* < −0.4 or lower), which indicate that the fitting of one metabolite influences the other. Metabolite ratios scaled to tCr and concentrations scaled to water are reported. Ratios over tCr are reported as normalized to 8 mM tCr. To obtain water scaled concentrations, water reference spectrum obtained in the same VOI as water-suppressed metabolite spectrum was used (Ernst et al., 1993; Kreis et al., 1993). Concentrations were corrected for T_1_ and T_2_ relaxation effects of tissue water. VOI composition was assumed to consist entirely of gray matter, with a tissue water fraction of 77% (Leithner et al., 2010). T_1_ and T_2_ relaxation times were set to 1,700 ms (Guilfoyle et al., 2003) and 50 ms (Li et al., 2016), respectively.

Statistical comparisons between HA and SL groups, as well as across post-injury time points, were conducted using two-tailed unpaired *t*-tests with unequal variance. Since all animals were alive for HA-SL comparisons, we also performed an additional repeated measures ANOVA (rmANOVA) to measure how the two trend lines differ. For mTBI, we first compared the differences between the baseline and different time points after CHI for pooled dataset. We then compared HA and SL groups at each time points. Given the pilot nature of the study, unpaired t-tests were used for exploratory comparisons, while rmANOVA was used to assess longitudinal group effects.

Statistical analyses were performed using MATLAB. For longitudinal comparisons between SL and HA groups during the 12-week adaptation phase, a Repeated Measures ANOVA (rmANOVA) was employed to assess group-by-time interactions, as this model accounts for the correlation between repeated measurements on the same animal.

For the post-mTBI phase, where mortality resulted in varying group sizes and compromised the assumptions of rmANOVA, two-tailed unpaired *t*-tests with Welch’s correction (accounting for unequal variance) were used for cross-sectional comparisons at specific time points (Day 3 and Day 14). While we acknowledge that *t*-tests in a small cohort increase the risk of Type II errors, they were selected as the most transparent method for identifying robust metabolic shifts in this exploratory pilot study. Significance was defined at *p* < 0.05. Due to the pilot nature of the study, *p*-values were not adjusted for multiple comparisons to avoid over-masking potentially significant biological trends that warrant further investigation in larger cohorts.

## Results

### Physiological and behavioral validation of the model

To ensure the MRS data reflects a translationally relevant model of HA-mTBI, we verified the physiological and behavioral status of the animal cohorts. Consistent with the hematological adaptations observed in chronic hypobaric hypoxia, mice exposed to 12 weeks of HA exhibited characteristic acclimatization responses prior to injury (Cramer et al., 2019). In parallel behavioral assessments conducted in this model, HA exposure alone was sufficient to induce robust impairments in contextual fear memory recall compared to SL controls injury (Cramer et al., 2019). Following the induction of mTBI via closed head injuries, animals exhibited acute righting reflex delays indicative of loss of consciousness, confirming the delivery of a mild concussive injury consistent with established protocols. White matter was significantly impacted with increased trace, radial diffusivity, and T_2_ values in the HA-mTBI. Previous volumetric analysis in similar cohorts revealed that while HA exposure initially increased global brain volume likely due to vasogenic edema, the superimposition of mTBI resulted in a subsequent reduction in brain volume and region-specific atrophy (Browne et al., 2026). The following ^1^H-MRS results must therefore be interpreted within the context of an HA adaptively stressed, edematous, and cognitively impaired brain during final stages of maturation, when brain undergoes structural and metabolic refinements between the ages of 8 weeks and 4 months.

### Animal cohort and data availability

All mice successfully underwent simulated HA exposure. We initially acquired longitudinal datasets at weeks 0, 4, and 12 from cohorts of *N* = 5 HA and *N* = 5 SL mice. However, two mice died following 3 episodes of CHI for mTBI induction, and two addition mice died by week 14. To maintain statistical power, data from two additional SL mice (available from weeks 12 to 14) were incorporated, and the two mice that did not survive the CHI procedures were excluded from the mTBI analysis. As a result, sample sizes for mTBI analysis were *N* = 4 HA and *N* = 6 SL at week 12 (for both pre- and post-mTBI), and *N* = 3 HA and *N* = 5 SL for week 14.

Following the screening of each acquired dataset, datasets with water linewidths exceeding 20 Hz were excluded as they were deemed to have poor B_0_ shimming. This resulted in the exclusion of 28 out of 207 metabolite datasets in the final analysis. In addition, Gln fitting results were found to be unreliable in the right hippocampus (CRLB > 20%), so the sum of Glu and Gln (denoted as Glx) is reported instead. Summary metrics for linewidth, signal-to-noise ratio, and CRLB of key metabolites are presented in Figures 1B,C shows representative VOI placements and their corresponding spectra.

Moderate to strong negative correlations were observed between several metabolite pairs: NAA and NAAG (*r* = −0.4 to −0.5), Cr and PCr (*r* = −0.6 to −0.7), PCho and GPC (*r* = −0.6 to −0.8), and Glc and Tau (*r* = −0.5 to −0.7). Consequently, these pairs are reported as summed concentrations: total NAA (tNAA), total creatine (tCr), total choline (tCho), and Glc + Tau.

In summary, the concentrations are reported for Glu, Gln, Ins, tNAA, tCr, tCho, and Glc + Tau. For the right hippocampus specifically, Glx is reported in place of Gln. The same convention applies to ratios normalized to tCr: Glu/tCr, Gln/tCr (except right hippocampus), Glx/tCr (right hippocampus only), Ins/tCr, tNAA/tCr, tCho/tCr, and (Glc + Tau)/tCr.

### Comparing HA and SL measurements

**FIGURE 2:**
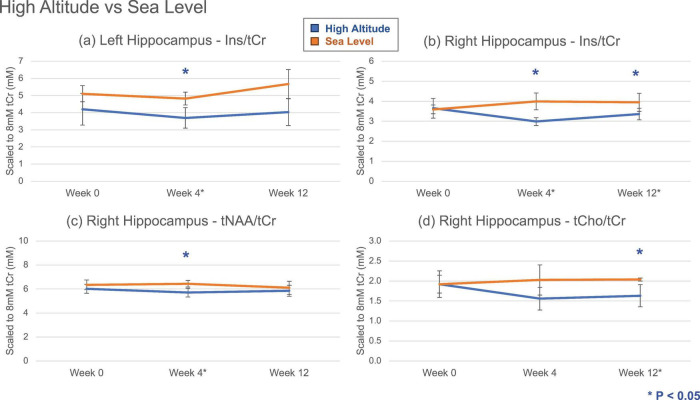
Mean metabolite levels and standard deviations (ratio to tCr, normalized to 8 mM) across time points for high altitude (HA, *N* = 5) and sea level (SL, *N* = 5) groups (same animals for all time points). Significant group differences (*P < 0.05; 2-tailed, unpaired *t*-test with unequal variance) were observed for Ins in both hippocampi **(a,b)**, and for tNAA and tCho in the right hippocampus **(c,d)**. Some metabolite alterations appeared transient, while others persisted beyond 12 weeks. Ins, myo-Inositol; tNAA, total NAA (sum of NAA and NAAG); tCr, total creatine (sum of Cr and PCr); tCho, total choline (sum of PCho and GPC)

Significant differences in Ins/tCr levels between the HA and SL groups were observed in both hippocampal formations (Figure 2). The right hippocampus also exhibited significant (*P* < 0.05) group differences in tNAA/tCr and tCho/tCr. In all of those cases, HA group exhibited lower tCr ratio compared to SL groups. Furthermore, rmANOVA revealed that Ins/tCr trendlines in right hippocampus differed significantly between the HA and SL groups (Supplementary Table S1).

Regarding estimated concentrations (Supplementary Table S2), significant HA and SL group differences were again observed for Ins levels in the right hippocampus in both *t*-test and rmANOVA with lowered levels in HA. Notably, we observed divergent trends in the frontal cortex where the metabolite levels generally increased in the SL group but decreased in the HA group. Additionally, tCr levels in the left hippocampus showed significant differences between groups with a higher value in HA group.

### Comparing the effect of mTBI on HA and SL groups

**FIGURE 3:**
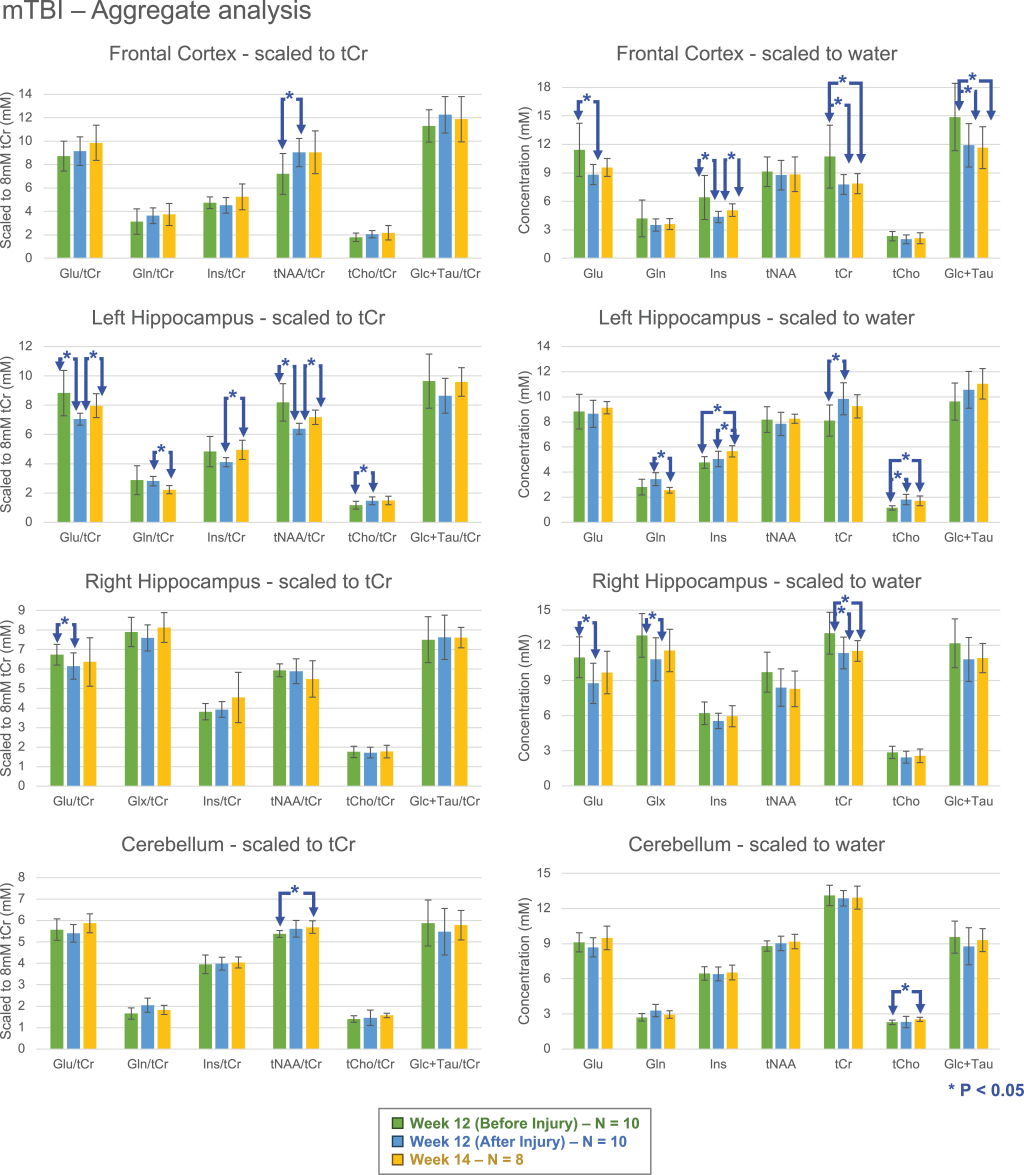
Mean metabolite levels and standard deviations (left column, ratio to tCr, normalized to 8 mM; right column, concentrations) for Glu, Gln/Glx, Ins, tNAA, tCr, tCho, and Glc + Tau at various time points following mTBI, aggregating data from both high altitude (HA) and sea level (SL) groups. Significant differences (*P < 0.05; 2-tailed, unpaired *t*-test with unequal variance) were observed between pre- and post-injury groups across brain regions, with tNAA/tCr showing the most pronounced change. mTBI, mild traumatic brain injury; Glu, glutamate; Gln, glutamine; Glx, sum of glutamine and glutamate; Ins, myo-Inositol; tNAA, total NAA (sum of NAA and NAAG); tCr, total creatine (sum of Cr and PCr); tCho, total choline (sum of PCho and GPC); Glc + Tau, sum of glucose and taurine.

Post-mTBI neurochemical profiles of all animals (combined pool of HA and SL) revealed the tNAA/tCr ratio, a biomarker of neuronal viability, as the most affected metabolite following CHI (Figure 3) with either significantly decreasing (between week 12 pre- and post-mTBI in left hippocampus) or increasing (between week 12 pre- and post-mTBI in frontal cortex; between week 12 post-mTBI and week 14 in left hippocampus; between week 12 pre-mTBI to week 14 in cerebellum). However, no significant changes were observed in tNAA levels when analyzed using concentration estimates. Instead, estimated tCr levels changed significantly after CHI in all regions except the cerebellum (decreased between week 12 pre- and post-mTBI in frontal cortex and right hippocampus and increased in between week 12 pre- and post-mTBI in left hippocampus). The increase in tNAA/tCr following CHI primarily reflects reductions in tCr rather than increases in estimated tNAA. Certain post-CHI alterations, such as changes in Ins and tCho in the left hippocampus, were consistent across both tCr ratios and estimated concentrations.

**FIGURE 4:**
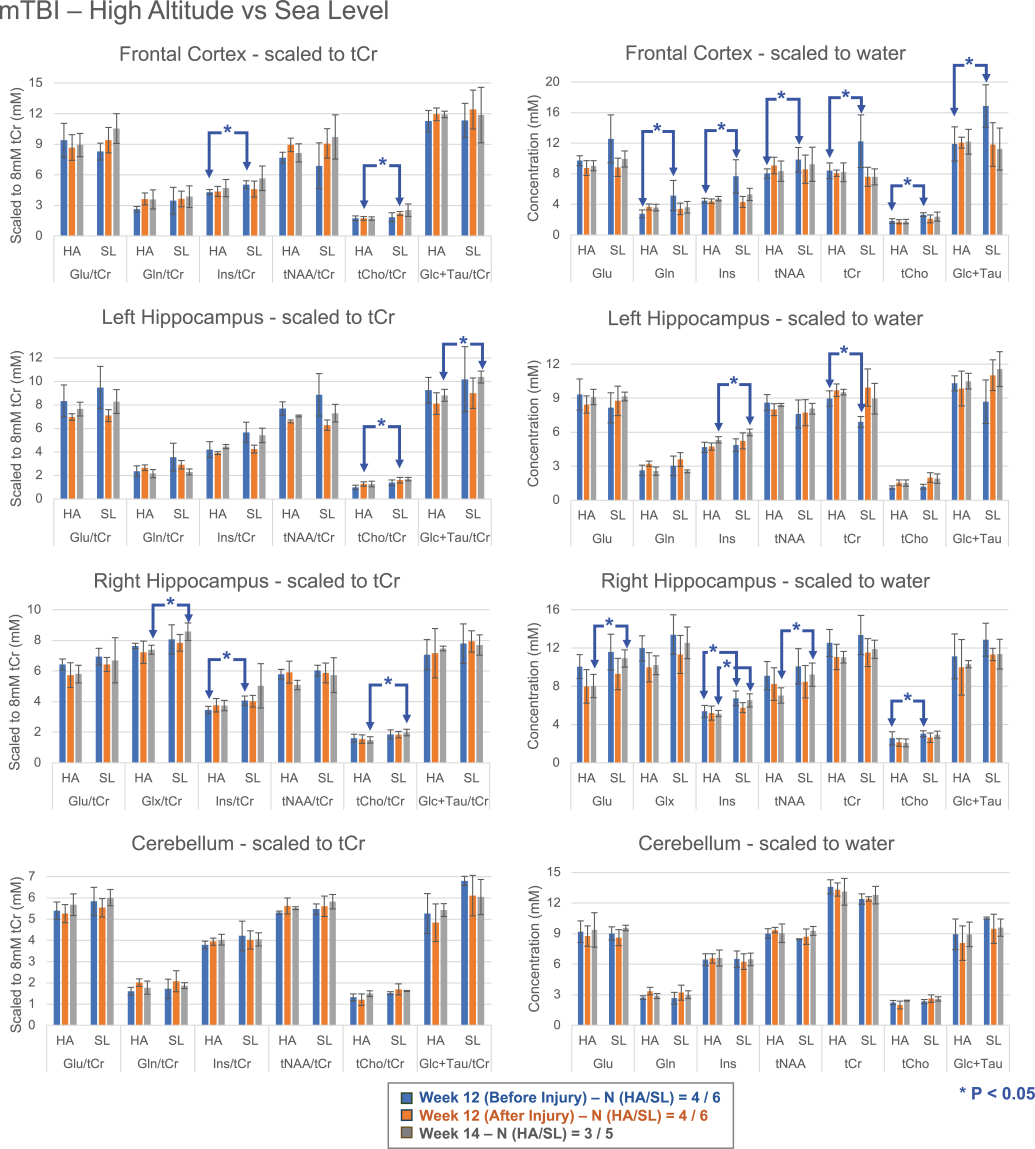
Mean metabolite levels and standard deviations (left column—ratio to tCr, normalized to 8 mM; right column –concentration) for Glu, Gln/Glx, Ins, tNAA, tCr, tCho and Glc + Tau at various time points following mTBI for HA and SL groups. Significant differences (*P < 0.05; 2-tailed, unpaired *t*-test with unequal variance) were observed between pre- and post-injury groups across brain regions, with tCho showing the most pronounced group differences. Note that the pool of animals is different compared to the previous Figure 3, accounting for the differences between the two figures. mTBI, mild traumatic brain injury; HA, high altitude; SL, sea level; Glu, glutamate; Gln, glutamine; Glx, sum of glutamine and glutamate; Ins, myo-Inositol; tNAA, total NAA (sum of NAA and NAAG); tCr, total creatine (sum of Cr and PCr); tCho, total choline (sum of PCho and GPC); Glc + Tau, sum of glucose and taurine.

Cross-sectional comparisons between the HA and SL groups (Figure 4) indicated that tCho was the most prominently affected metabolite post-mTBI, showing significant group differences (lower levels in HA) in both tCr ratios and concentration estimates across all regions except the cerebellum. Ins levels also differed significantly in both hippocampi with lower levels in HA. Consistent with the earlier pre-mTBI HA-SL comparisons, estimated concentrations in the frontal cortex showed significant group differences at pre-CHI timepoints. However, these differences diminished in the subsequent post-CHI measurements.

## Discussion

In this study, we exposed a small cohort of young adult (8 weeks old) mice to simulated HA conditions and monitored neurochemical changes over 12 weeks across four distinct brain regions using ^1^H MRS. Subsequently, we performed CHI to evaluate how HA-preconditioned brains respond differently to mTBI compared to SL controls for another 2 weeks. Despite the pilot nature of this study, we identified significant differences in neurochemical levels between the HA and SL groups at multiple time points in multiple brain regions.

### Changes seen in chronic HA exposure: impact on metabolic refinements during the final stages of maturation under hypoxia

Comparison between the HA and SL groups during chronic exposure revealed that Ins/tCr and Ins concentrations in the right hippocampus were significantly lower in the HA group compared to SL controls. Certain alterations, such as Ins/tCr in the left hippocampus and tNAA in the right hippocampus, appeared transient. In contrast, others, such as tCr in the left hippocampus and tCho/tCr in the right hippocampus, persisted beyond 12 weeks. Ins is a primary organic osmolyte and a marker of glial cell density and activation.

The observed reduction in Ins likely represents a compensatory metabolic adaptation to chronic hypoxic exposure. Our previous morphological analysis in this model demonstrated increased global brain volume and alterations in T_2_ relaxation times at 12 weeks of HA (Cramer et al., 2019), indicative of sustained vasogenic edema and fluid shifts. In the setting of cerebral edema, brain cells actively deplete intracellular organic osmolytes, such as Ins, to prevent excessive cellular swelling and maintain volume homeostasis (Govindaraju et al., 2000; Hackett et al., 2019). Therefore, the lower Ins levels in the HA group likely reflect this osmotic downregulation in response to the chronic vascular leak and brain swelling previously characterized in similar cohorts.

Similarly, the reduction in the tCho/tCr ratio observed in the HA group parallels the findings for Ins/tCr and further supports the hypothesis of an osmoregulatory adaptation to chronic hypoxia. The ^1^H-MRS choline peak comprises GPC and PCho, which are primary markers of membrane phospholipid metabolism and turnover (Ross and Bluml, 2001). Like Ins, GPC serves as a significant organic osmolyte in neural tissue. The concurrent reduction in tCho likely reflects the cellular extrusion of organic osmolytes to counteract osmotic swelling and preserve cellular integrity.

Notably, metabolite levels in the SL group fluctuated across time points. Given that the mice aged from 8 to 20 weeks during the chronic exposure, and their life expectancy being around 24–30 months, these fluctuations likely reflect ongoing neurodevelopment. Consequently, the relative lack of change observed in some measurements in the HA group may indicate a hypoxia-induced delay or altered metabolic trajectories relative to age-matched SL controls.

The significant reduction of Ins in the HA group likely represents a compensatory metabolic adaptation. As the brain acclimatizes to hypobaric hypoxia, it experiences sustained vasogenic edema. To prevent excessive cellular swelling, neural cells deplete organic osmolytes like Ins and GPC (tCho). This “osmoregulatory exhaustion” may leave the HA brain with less capacity to handle the acute ionic shifts triggered by a subsequent mTBI.

At week 12, the frontal cortex showed the most substantial reduction at HA compared to SL in concentrations across all metabolites consistent with findings reported in a pilot study by Prescot et al. (2021). However, because these differences were not mirrored in the tCr ratios, we suspect a systemic bias related to tissue water content. Since concentration estimation relies on the amplitude of reference water spectra, tissue water content and assumed T_1_/T_2_ parameters, hypoxia-induced changes in brain hydration may have influenced the quantification. Nevertheless, these alterations collectively suggest shifts in astroglial osmoregulation, membrane turnover, and neuronal metabolic stress, reflecting an energy-conservation phenotype under chronic hypobaric-hypoxia.

The divergent trends observed in the frontal cortex highlight a critical distinction between physical maturation and ongoing neurodevelopmental refinement. While the mice were physiologically mature at the start of the study (8 weeks), the SL group’s metabolic fluctuations likely reflect the final stages of brain development (Arellano et al., 2024; Chen et al., 2021; Kwong and Sohal, 2000). In contrast, the HA group’s declining or stagnant metabolite levels suggest that chronic hypoxia interferes with these developmental trajectories, forcing a shift toward an “energy-conservation phenotype” (Prescot et al., 2021). This pre-injury state of “metabolic fragility” likely underlies the severe bioenergetic failure observed following the subsequent mTBI.

Furthermore, the observation of more pronounced metabolic shifts in the right hippocampus—specifically the significant group differences in Ins, tNAA, and tCho ratios—suggests a lateralized vulnerability to chronic hypoxic stress. This asymmetry may stem from vascular, differences in the circle of Willis and/or anterior/posterior cerebral and communicating arteries (McColl et al., 2004) in addition to regional variations in blood-brain barrier permeability. Under the extreme physiological demand of chronic hypobaric hypoxia, these baseline asymmetries can be amplified, leading one hemisphere to reach a “etabolic tipping point” or a state of “osmoregulatory exhaustion” more rapidly than the other (Vagnozzi et al., 2007) Prescot et al. (2021). This unilateral vulnerability underscores that the brain’s adaptation to altitude is not a uniform process, and standard bilateral assessments may miss localized neurochemical failures that precede structural atrophy.

### Changes seen in mTBI

When aggregating all animals, post-CHI measurements (week 12–14) showed marked increases in tNAA/tCr ratios and decreases in estimated tCr levels, consistent with established literature on traumatic injury (Signoretti et al., 2010; Vagnozzi et al., 2013). The frontal cortex and bilateral hippocampi showed the most dramatic fluctuations. Therefore, the observed aggregate reduction in estimated concentrations of Glu, tCr, and Glc + Tau post-mTBI in frontal cortex is indicative of a generalized state of metabolic depression. Specifically, the depletion of the creatine pool aligns with the “window of metabolic brain vulnerability,” suggesting an impairment in the brain’s capacity to buffer ATP energy stores or a disruption in creatine synthesis and transport during the acute post-injury phase (Shaw, 2002). The depletion of the total creatine pool post-injury may be exacerbated by systemic glucose competition. Martí-Mateos et al. (2026) demonstrated that HA (simulated by moderate hypoxia 11% O_2_)-induced erythrocytosis creates a massive glucose sink in the blood, which could limit substrate availability for the injured brain during its window of metabolic vulnerability. Furthermore, these broad neurochemical reductions likely reflect the downstream consequences of mitochondrial dysfunction and bioenergetic failure, which we and others have previously characterized as a hallmark of the subacute response to concussive injury.

When disaggregating the groups, tCho emerged as the most profound differentiator post-injury, particularly in the frontal cortex and hippocampi. We also observed significant group differences in Ins concentrations within the hippocampi. Because choline and Ins are integral to membrane integrity and glial signaling, the reduced levels in HA mice suggests these cells are becoming “xhausted” or overtaxed. These results are significant as Ins supplementation has demonstrated neuroprotective effects and reduced cell death in models of epilepsy and chemical insult (Kandashvili et al., 2022; Tsverava et al., 2016), suggesting its potential utility as a therapeutic intervention for mTBI. Furthermore, at 14 days post-injury, the SL group exhibited higher tNAA levels in the right hippocampus. As NAA is a primary marker of neuronal viability, this suggests superior neuronal recovery or preservation in animals maintained at sea level. The reduction in tNAA is a hallmark of neuronal metabolic crisis, as NAA synthesis is directly coupled to mitochondrial function and the availability of acetyl-CoA (Moffett et al., 2013). The failed recovery of tNAA in HA mice suggests that chronic neuroinflammation stalls neuronal repair. Hatch et al. (2024) found that HA-activated microglia release cytokines that inhibit hippocampal long-term potentiation, providing a functional mechanism for the persistent metabolic depression we observed via ^1^H-MRS. This decline parallels the reductions in cerebral glucose metabolism (we previously detected via PET imaging in the ipsilateral cortex (Browne et al., 2026)). In the context of HA, where baseline oxidative phosphorylation is already challenged, the metabolic demand of repairing TBI-induced damage likely outstrips ATP production, mirroring the “metabolic crisis” described by Vagnozzi et al. (2007). The persistence of altered tNAA in the HA group suggests that the hypoxic environment impairs the restoration of acetyl-CoA pools required for NAA resynthesis and myelin repair.

The persistent depression of tNAA in the HA group 14 days post-mTBI—when the SL group had begun to recover—suggests that the baseline hypoxic stress impairs the resynthesis of acetyl-CoA. This indicates that at high altitude, a ‘mild’ injury can trigger a “severe” metabolic crisis due to depleted ATP buffering capacity, represented by the reduced tCr pool.

### Limitations and future work

Several limitations in the current study warrant consideration. First, as a pilot investigation, the sample size was relatively small (5 per group initially) and utilized only male mice. While the use of 7 T ^1^H-MRS provided high sensitivity, the modest cohort size and subsequent post-mTBI mortality may limit the statistical power to detect more subtle neurochemical alterations and increases the risk of Type II errors. A larger, more comprehensive study will be necessary to validate these preliminary findings.

Second, our quantification relied on several physiological assumptions, including fixed T_1_/T_2_ times and tissue water fractions. Given that high-altitude exposure is known to alter brain hydration and tissue properties, these fixed parameters may introduce systematic biases in concentration estimates. Further work that incorporates more detailed characterization of tissue properties and acquisition of an actual macromolecular spectra could improve the accuracy of MRS quantification.

Third, while myo-inositol and total choline are established markers of osmoregulation and glial activity, ^1^H-MRS cannot distinguish between specific glial subtypes (e.g., astrocytes vs. microglia). Therefore, the neurochemical shifts observed cannot be definitively linked to a single cellular process without further immunohistochemical validation.

Finally, spectral quality could be further enhanced by using advanced MRS protocols (Wilson et al., 2019) that reduce chemical shift displacement errors at 7T, such as LASER and semi-LASER pulse sequences. Improved signal quality would enable more precise characterization of neurochemical changes and potentially increase the number of reliably quantifiable metabolites.

## Conclusion

The study identified region-specific neurochemical changes in mice following HA exposure and mTBI. Significant alterations in Ins, tNAA, and tCho suggest that chronic HA exposure significantly modifies the brain’s metabolic environment and its subsequent response to head trauma.

Crucially, these data indicate that the HA-acclimatized brain is not metabolically equivalent to the SL brain when sustaining traumatic insult. The observed decoupling of neurochemical indices (tCho, tNAA) from the persistent vascular and structural deficits noted in our previous study suggests that standard “return-to-duty” or “return-to-play” protocols derived from sea-level norms may be insufficient for personnel operating at altitude. While clinical symptoms may resolve, ^1^H-MRS provides a non-invasive window into the underlying “invisible” metabolic injury that often persists beyond functional recovery suggesting that HA-adopted brains exhibit decoupling of symptoms and metabolism and prolonged “metabolic crisis.” Future therapeutic strategies should specifically target this bioenergetic deficit, potentially via metabolic substrates that bypass impaired glycolytic or mitochondrial pathways, to mitigate the cumulative risk of long-term neurodegeneration in high-altitude environments.

While these findings highlight potential biomarkers for altitude-related neurological vulnerability, the modest sample size necessitate further preclinical investigation analysis with expanded cohorts to establish clear profiles of the neurochemical biomarkers in HA adapted brains following mTBI. Future clinical research should prioritize the development of metabolic-based “return-to-duty” protocols that utilize ^1^H-MRS to identify “invisible” neurochemical vulnerabilities in populations operating in extreme environments.

## Data Availability

The raw data supporting the conclusions of this article will be made available by the authors, without undue reservation.

## Author disclaimer

This research was conducted while Asamoah Bosomtwi, Ph.D., and Nathan P. Cramer, Ph.D., were employed by the Henry M. Jackson Foundation for the Advancement of Military Medicine, Inc. in support of the Department of Radiology and Bioengineering and the Department of Anatomy and Neurobiology, respectively, at the Uniformed Services University of the Health Sciences. Their contributions to this work were limited in scope at their current affiliations: Augusta University and the University of Maryland School of Medicine. The opinions and assertions expressed herein are those of the author(s) and do not reflect the official policy or position of the Uniformed Services University of the Health Sciences, the Department of War, or any other agency of the U.S. Government. The opinions and assertions contained herein are those of the author(s) and not to be construed as reflecting the views of the Henry M. Jackson Foundation for the Advancement of Military Medicine Inc.
