# Supplementary material for: 1H-MRS brain metabolites as biomarkers of high-altitude hypobaric hypoxia following mild traumatic brain injury in mice
Source: Front Neurosci. 2026 May 14;20:1808567. doi: 10.3389/fnins.2026.1808567 (PMC13181442; doi:10.3389/fnins.2026.1808567)
Supplement: Supplementary file 1 [file Table_1.pdf]

Summary tCr Ratio		FC			HL			HR			CB		
		Week 0	Week 4	Week 12	Week 0	Week 4	Week 12	Week 0	Week 4	Week 12	Week 0	Week 4	Week 12
**Glu/tCr**	**HA**	8.6 (±2.4)	8.7 (±1.2)	9.0 (±1.0)	8.7 (±0.6)	7.9 (±0.4)	8.2 (±1.2)	7.4 (±0.3)	5.7 (±0.2)	6.3 (±0.5)	5.7 (±0.4)	5.7 (±0.4)	5.3 (±0.4)
	**SL**	8.9 (±1.5)	8.8 (±1.9)	8.3 (±1.0)	8.5 (±0.4)	8.3 (±0.6)	10.2 (±1.5)	7.3 (±0.4)	6.7 (±0.6)	6.7 (±0.3)	5.4 (±0.2)	5.7 (±0.6)	5.5 (±0.1)
**Gln/tCr (Glx/tCr for HR)**	**HA**	4.0 (±2.2)	4.1 (±2.9)	3.7 (±1.8)	3.3 (±1.6)	3.4 (±2.0)	3.1 (±2.1)	8.8 (±0.2)	7.5 (±1.5)	8.1 (±1.5)	2.5 (±1.7)	4.1 (±2.9)	2.7 (±2.0)
	**SL**	2.9 (±0.7)	3.9 (±0.9)	3.7 (±1.3)	2.4 (±0.7)	2.8 (±0.8)	3.9 (±1.2)	8.0 (±0.8)	7.6 (±0.2)	7.7 (±0.9)	1.9 (±0.2)	1.9 (±0.2)	1.5 (±0.1)
**Ins/tCr**	**HA**	5.3 (±0.6)	4.6 (±0.5)	4.4 (±0.3)	4.2 (±0.9)	**3.7 (±0.6)**	4.0 (±0.8)	3.6 (±0.5)	**3.0 (±0.2)**	**3.4 (±0.3)**	3.9 (±0.8)	3.3 (±1.4)	3.4 (±0.8)
	**SL**	4.6 (±1.0)	5.7 (±1.7)	4.8 (±0.4)	5.1 (±0.5)	**4.8 (±0.4)**	5.7 (±0.8)	3.6 (±0.2)	**4.0 (±0.4)**	**3.9 (±0.5)**	4.3 (±0.3)	4.2 (±0.3)	3.8 (±0.1)
**tNAA/tCr**	**HA**	7.8 (±1.1)	8.4 (±2.2)	8.1 (±1.1)	7.4 (±0.4)	7.1 (±0.4)	7.7 (±0.6)	6.0 (±0.4)	**5.7 (±0.4)**	5.9 (±0.5)	5.4 (±0.3)	5.5 (±0.0)	5.4 (±0.1)
	**SL**	7.8 (±0.4)	8.9 (±2.7)	7.4 (±1.8)	7.5 (±0.4)	7.6 (±0.8)	9.3 (±1.7)	6.4 (±0.4)	**6.4 (±0.3)**	6.1 (±0.5)	5.2 (±0.4)	5.3 (±0.3)	5.5 (±0.3)
**tCho/tCr**	**HA**	2.0 (±0.1)	1.5 (±0.3)	1.6 (±0.2)	1.6 (±0.3)	1.0 (±0.3)	1.1 (±0.2)	1.9 (±0.3)	1.6 (±0.3)	**1.6 (±0.3)**	1.7 (±0.2)	1.4 (±0.2)	1.5 (±0.1)
	**SL**	2.0 (±0.2)	2.0 (±0.4)	1.9 (±0.4)	1.3 (±0.2)	1.2 (±0.2)	1.2 (±0.5)	1.9 (±0.2)	2.0 (±0.4)	**2.0 (±0.0)**	1.8 (±0.3)	1.6 (±0.1)	1.4 (±0.2)
**Glc+Tau/tCr**	**HA**	12.4 (±3.3)	12.0 (±1.6)	12.0 (±0.9)	9.2 (±1.8)	9.1 (±1.5)	8.9 (±1.3)	7.5 (±1.0)	7.3 (±0.7)	7.4 (±0.8)	**6.2 (±0.6)**	5.6 (±0.1)	5.6 (±0.8)
	**SL**	12.6 (±2.3)	12.3 (±4.7)	11.8 (±1.3)	10.8 (±1.5)	9.8 (±1.0)	11.3 (±3.3)	7.2 (±1.1)	7.1 (±0.6)	7.9 (±1.4)	**7.6 (±0.9)**	5.8 (±0.7)	6.9 (±0.3)
**p-value (unpaired 2-tailed t-test)**	**Glu**	0.830	0.937	0.243	0.525	0.293	0.137	0.727	0.058	0.215	0.134	0.878	0.616
	**Gln / Glx**	0.326	0.876	0.961	0.290	0.595	0.529	0.075	0.968	0.638	0.483	0.466	0.307
	**Ins**	0.220	0.230	0.083	0.101	**0.011**	0.053	0.843	**0.011**	**0.046**	0.389	0.504	0.337
	**tNAA**	0.986	0.783	0.501	0.653	0.284	0.226	0.236	**0.010**	0.462	0.591	0.368	0.646
	**tCho**	0.802	0.068	0.187	0.110	0.247	0.710	0.999	0.090	**0.031**	0.781	0.406	0.954
	**Glc+Tau**	0.927	0.873	0.788	0.164	0.466	0.324	0.715	0.746	0.480	**0.047**	0.591	0.061
**p-value (rmANOVA)**	**Glu**	0.598			0.164			0.132			0.350		
	**Gln / Glx**	0.825			0.816			0.571			0.661		
	**Ins**	0.216			0.060			**0.014**			0.633		
	**tNAA**	0.818			0.147			0.090			0.437		
	**tCho**	0.063			0.149			0.055			0.983		
	**Glc+Tau**	0.957			0.427			0.672			0.357		

$p < 0.05$

Supporting Table ST1. Mean metabolite levels and standard deviations (ratio to tCr, normalized to 8 mM), shown as mean (± standard deviation), across time points for high altitude (HA, N = 5) and sea level (SL, N = 5) groups (same animals for all time points) as well as detailed results of the statistical analysis are shown. Blue numbers denote metabolite pairing and p-values that show significance ($p < 0.05$).

Abbreviations: rmANOVA – repeated measures analysis of variance; Wk – week; FC – frontal cortex; HL – left hippocampus; HR – right hippocampus; CB – cerebellum; Glu – glutamate; Gln – glutamine; Glx – sum of glutamine and glutamate; Ins – myo-Inositol; tNAA – total NAA (sum of NAA and NAAG); tCr – total creatine (sum of Cr and PCr); tCho – total choline (sum of PCho and GPC); Glc+Tau – sum of glucose and taurine

Summary Concentration		FC			HL			HR			CB		
		Week 0	Week 4	Week 12	Week 0	Week 4	Week 12	Week 0	Week 4	Week 12	Week 0	Week 4	Week 12
Glu	HA	9.7 (±0.9)	9.4 (±1.7)	**9.3 (±0.2)**	**8.7 (±0.6)**	7.9 (±0.5)	8.7 (±1.3)	13.3 (±1.4)	8.7 (±1.6)	9.9 (±2.2)	8.3 (±0.9)	8.6 (±1.5)	8.3 (±1.0)
	SL	10.3 (±1.5)	9.9 (±3.0)	**12.2 (±2.0)**	**7.8 (±0.3)**	7.9 (±0.6)	9.0 (±0.9)	13.4 (±1.8)	10.5 (±2.8)	11.2 (±1.7)	7.3 (±1.7)	8.8 (±0.7)	8.8 (±0.3)
Gln (Glx for HR)	HA	5.6 (±5.5)	4.1 (±2.0)	3.8 (±1.6)	3.3 (±1.8)	3.4 (±2.0)	3.2 (±1.8)	15.8 (±1.8)	11.3 (±2.1)	12.3 (±1.1)	3.7 (±2.6)	6.4 (±4.9)	4.2 (±2.8)
	SL	3.6 (±1.6)	4.7 (±2.5)	5.5 (±2.0)	2.2 (±0.7)	2.6 (±0.7)	3.5 (±1.0)	14.5 (±1.8)	11.7 (±2.1)	12.9 (±1.8)	2.6 (±0.4)	2.9 (±0.4)	2.4 (±0.3)
Ins	HA	6.6 (±3.2)	5.0 (±1.5)	**4.5 (±0.4)**	4.2 (±0.9)	3.7 (±0.6)	4.3 (±1.0)	6.5 (±0.3)	**4.5 (±0.7)**	**5.2 (±0.9)**	5.6 (±1.0)	4.8 (±1.6)	5.3 (±1.4)
	SL	5.3 (±0.8)	6.3 (±1.9)	**7.1 (±1.0)**	4.7 (±0.3)	4.6 (±0.6)	5.0 (±0.4)	6.6 (±0.8)	**6.2 (±1.1)**	**6.6 (±0.5)**	5.8 (±1.0)	6.6 (±0.2)	6.2 (±0.3)
tNAA	HA	9.1 (±2.0)	8.9 (±1.6)	**8.4 (±0.7)**	7.4 (±0.6)	7.1 (±0.4)	8.1 (±0.5)	10.8 (±1.0)	8.6 (±1.5)	9.2 (±2.1)	7.8 (±0.8)	8.2 (±0.8)	8.3 (±0.5)
	SL	9.2 (±2.3)	9.7 (±2.2)	**10.7 (±0.9)**	6.9 (±0.5)	7.2 (±0.6)	8.3 (±1.1)	11.7 (±1.7)	10.0 (±1.9)	10.4 (±2.7)	7.1 (±1.1)	8.3 (±0.3)	8.8 (±0.6)
tCr	HA	9.7 (±3.6)	9.0 (±3.2)	**8.3 (±0.9)**	8.0 (±1.0)	8.0 (±0.8)	**8.5 (±0.7)**	14.4 (±1.7)	12.1 (±1.9)	12.4 (±2.0)	11.6 (±0.8)	12.0 (±1.2)	12.4 (±1.0)
	SL	9.5 (±2.7)	9.9 (±5.3)	**12.0 (±2.4)**	7.3 (±0.3)	7.6 (±0.6)	**7.2 (±0.5)**	14.7 (±2.2)	12.4 (±2.3)	13.5 (±2.3)	10.9 (±2.2)	12.5 (±0.5)	12.8 (±0.2)
tCho	HA	2.4 (±0.9)	1.6 (±0.3)	**1.7 (±0.3)**	1.6 (±0.5)	1.0 (±0.5)	1.2 (±0.2)	3.5 (±0.8)	2.4 (±0.7)	2.6 (±0.8)	2.5 (±0.4)	2.1 (±0.5)	2.3 (±0.2)
	SL	2.4 (±0.5)	2.4 (±0.9)	**2.8 (±0.2)**	1.2 (±0.2)	1.1 (±0.2)	1.1 (±0.4)	3.5 (±0.3)	3.2 (±1.0)	3.4 (±0.6)	2.3 (±0.3)	2.5 (±0.3)	2.3 (±0.2)
Glc+Tau	HA	14.0 (±2.2)	12.9 (±2.8)	**12.5 (±1.5)**	9.0 (±1.3)	9.1 (±1.1)	9.5 (±1.8)	13.4 (±1.6)	11.1 (±2.1)	11.4 (±1.9)	8.9 (±0.7)	8.3 (±0.7)	8.8 (±1.6)
	SL	14.4 (±1.8)	13.1 (±2.5)	**17.4 (±2.2)**	9.9 (±1.4)	9.2 (±0.4)	10.1 (±2.7)	13.0 (±0.6)	11.1 (±2.3)	13.2 (±2.4)	10.1 (±1.1)	9.0 (±0.8)	11.0 (±0.6)
p-value (unpaired 2-tailed t-test)	Glu	0.492	0.735	**0.031**	**0.028**	0.904	0.673	0.928	0.315	0.310	0.356	0.861	0.452
	Gln / Glx	0.464	0.685	0.158	0.260	0.476	0.778	0.310	0.783	0.560	0.413	0.500	0.305
	Ins	0.422	0.283	**0.003**	0.272	0.073	0.196	0.854	**0.049**	**0.023**	0.832	0.354	0.306
	tNAA	0.948	0.553	**0.002**	0.235	0.824	0.800	0.382	0.301	0.440	0.306	0.918	0.418
	tCr	0.942	0.743	**0.023**	0.213	0.361	**0.019**	0.821	0.863	0.448	0.564	0.641	0.458
	tCho	0.931	0.143	**0.001**	0.118	0.581	0.785	0.969	0.235	0.100	0.535	0.486	0.780
	Glc+Tau	0.776	0.920	**0.004**	0.361	0.846	0.755	0.712	0.999	0.219	0.121	0.355	0.071
p-value (rmANOVA)	Glu	0.056			0.709			0.509			0.464		
	Gln / Glx	0.567			0.632			0.742			0.680		
	Ins	**0.009**			0.274			**0.006**			0.536		
	tNAA	**0.040**			0.540			0.490			0.142		
	tCr	0.145			**0.009**			0.910			0.487		
	tCho	**0.004**			0.827			0.234			0.881		
	Glc+Tau	**0.020**			0.782			0.721			0.204		

$p < 0.05$

Supporting Table ST2. Mean metabolite levels and standard deviations, shown as mean (± standard deviation), across time points for high altitude (HA, N = 5) and sea level (SL, N = 5) groups (same animals for all time points) as well as detailed results of the statistical analysis are shown. Blue numbers denote metabolite pairing and p-values that show significance ($p < 0.05$). Abbreviations: rmANOVA – repeated measures analysis of variance; Wk – week; FC – frontal cortex; HL – left hippocampus; HR – right hippocampus; CB – cerebellum; Glu – glutamate; Gln – glutamine; Glx – sum of glutamine and glutamate; Ins – myo-Inositol; tNAA – total NAA (sum of NAA and NAAG); tCr – total creatine (sum of Cr and PCr); tCho – total choline (sum of PCho and GPC); Glc+Tau – sum of glucose and taurine
